# Outpatient Waiting Time and Its Association With Patient Satisfaction and Willingness to Revisit a Tertiary Care Teaching Hospital in Jharkhand: A Cross-Sectional Study

**DOI:** 10.7759/cureus.116596

**Published:** 2026-09-20

**Authors:** Kritika Dev, Bhoopendra Singh, Shashi B Singh

**Affiliations:** 1 Hospital Administration, Rajendra Institute of Medical Sciences, Ranchi, IND; 2 Forensic Medicine and Toxicology, Rajendra Institute of Medical Sciences, Ranchi, IND; 3 Community Medicine, Rajendra Institute of Medical Sciences, Ranchi, IND

**Keywords:** consultation duration, outpatient department, patient flow, patient satisfaction, patient waiting time, revisit intention, tertiary care hospital

## Abstract

Background

Outpatient waiting is an important part of the patient experience and may influence satisfaction and continued use of health services. In high-volume tertiary hospitals, delays at successive service points can accumulate before clinical care begins. This study assessed waiting and consultation-time patterns, associated factors, patient satisfaction, and willingness to revisit a tertiary care teaching hospital in Jharkhand, India.

Methods

This observational cross-sectional study included 400 adults attending eight outpatient departments (OPDs) through convenience sampling. Registration waiting time, pre-consultation waiting time, doctor consultation duration, and total waiting time from registration to the start of consultation were reported using predefined ordered categories in a bilingual questionnaire. Patient characteristics, visit type, arrival time, satisfaction, perceived reason for waiting, and willingness to revisit were also recorded. Data were analyzed using frequencies, percentages, Pearson’s chi-square test, Cramér’s V, and Spearman’s rank correlation. A multivariable ordinal logistic regression model was used to assess whether total waiting time independently predicted poorer overall satisfaction. Monte Carlo significance estimates were used where small expected frequencies limited the suitability of asymptotic chi-square estimates. The Benjamini-Hochberg procedure was used to control the false discovery rate for exploratory department-stratified analyses.

Results

Among 400 participants, 193 (48.3%) were male participants and 207 (51.8%) were female participants. Registration waiting exceeded 60 minutes for 104 (26.0%) participants, while 119 (29.8%) waited more than 60 minutes before consultation. Most consultations, 323 (80.8%), lasted 30 minutes or less. Total waiting exceeded three hours for 233 (58.3%) participants. Total waiting time was significantly associated with visit type (χ²(3) = 51.096, p < 0.001; Cramér’s V = 0.357) and arrival time (χ²(9) = 179.485, p < 0.001; Cramér’s V = 0.387), but not with department. Longer registration, pre-consultation, and total waiting times were strongly associated with poorer corresponding satisfaction (Spearman’s ρ = 0.879, 0.871, and 0.816, respectively; all p < 0.001). Longer consultation duration was associated with better consultation satisfaction (ρ = -0.668, p < 0.001). After adjustment, each one-category increase in total waiting time was associated with higher odds of worse overall satisfaction (adjusted odds ratio (aOR) = 22.75, 95% CI: 13.68-37.83; p < 0.001). Total waiting time was strongly associated with willingness to revisit (χ²(6) = 249.166, p < 0.001; Cramér’s V = 0.558).

Conclusions

Prolonged outpatient waiting was common and was closely associated with poorer satisfaction and lower willingness to revisit. Visit type and arrival time appeared more relevant to total waiting than the demographic characteristics examined. Efforts to improve outpatient flow should focus on reducing avoidable registration and queue delays while preserving adequate doctor-patient consultation time.

## Introduction

Outpatient departments (OPDs) are an important point of entry to hospital care. For many patients, the experience of care begins well before they meet the doctor. Time spent at registration and in queues may influence how patients perceive the accessibility, organization, and quality of hospital services. In high-volume tertiary hospitals, delays at several stages can accumulate, resulting in a long interval between arrival and the beginning of clinical care.

Studies from Nigeria, Iran, and Ethiopia have documented substantial outpatient delays and have linked waiting time with factors such as patient volume, staffing, arrival patterns, and service organization [1-3]. Similar concerns have been reported in India. Langstieh et al. reported a mean waiting time of 110.86 minutes and a mean consultation duration of 5.34 minutes in a neurology OPD [4]. Studies from Gujarat, Maharashtra, northern India, and Uttarakhand have also described prolonged waiting in relation to comparatively shorter periods of direct clinical contact [5-8].

Waiting time is not simply an operational measure; it is also an important patient-centered outcome. Longer actual or perceived waiting has generally been associated with lower patient satisfaction [6,9-13]. However, this relationship may vary across healthcare settings, as differences in waiting duration do not always correspond to significant differences in overall satisfaction [14].

It is therefore useful to distinguish between time that patients spend waiting and time spent receiving clinical care. Improving efficiency should reduce avoidable delays without unnecessarily shortening consultations.

Although outpatient waiting is a key component of the patient experience, few studies in Jharkhand have evaluated distinct stages of the same outpatient journey alongside satisfaction and willingness to return.

The primary objective of this study was to quantify the distributions of registration waiting time, pre-consultation waiting time, doctor consultation duration, and total OPD waiting time among 400 adults attending eight OPDs of a tertiary care teaching hospital in Jharkhand, India, between March and July 2026.

The secondary objectives were to (i) compare these time-interval distributions across the eight participating departments; (ii) examine the associations of sociodemographic characteristics and visit-related operational characteristics with total OPD waiting time; (iii) assess the correlation between each waiting- or consultation-time measure and its corresponding patient-satisfaction outcome; and (iv) determine the association between total OPD waiting time and willingness to revisit the hospital for future outpatient services.

## Materials and methods

Study design and setting

This observational cross-sectional study was conducted from March to July 2026 in the OPD of Rajendra Institute of Medical Sciences, Ranchi, Jharkhand, India. It is a tertiary care teaching hospital with an average daily outpatient attendance of approximately 2,500-3,000 patients. Participants were recruited during routine working hours (9:00 AM-5:00 PM, Monday-Friday, and 9:00 AM-2:00 PM on Saturday) across eight clinical departments: Dermatology, ENT, Gynecology, Medicine, Neurology, Ophthalmology, Orthopedics, and Surgery.

The questionnaire was administered immediately after participants completed their consultation so that registration waiting time, pre-consultation waiting time, doctor consultation duration, and total OPD waiting time were captured for the same visit. To enable feasible, standardized data collection and comparative cross-departmental analysis, uniform time categories were applied across all specialties. However, it is recognized that these clinical departments operate under distinct workflow dynamics, service requirements, and patient complexity, ranging from rapid diagnostic sorting in high-volume clinics to time-intensive minor procedures or detailed neurological assessments. These operational variations were accounted for as contextual factors in data interpretation rather than adjusted within the measurement framework.

This report was prepared in accordance with the Strengthening the Reporting of Observational Studies in Epidemiology (STROBE) recommendations for cross-sectional studies [15].

Participants and sampling

Adults aged 20 years or older who had completed registration, waited for and completed their doctor consultation, could understand and respond to the study questions, and provided written informed consent were eligible for participation.

Patients requiring emergency care, those who were critically ill or unable to complete the routine OPD pathway, those with unavailable required information, and those receiving priority or expedited services outside the routine pathway were excluded.

Convenience sampling was used, and recruitment continued until 400 eligible and consenting participants had been enrolled. All enrolled participants had complete data and were included in the analysis. A pre-enrollment screening log was not maintained; therefore, the numbers of patients approached, assessed for eligibility, excluded, or declining participation were unavailable, and a true participation or response rate could not be calculated.

Sample size

The sample size was calculated using a single-proportion approach, assuming a prevalence of 50%, a 95% confidence level, and an absolute precision of 5% (the formula $n = \frac{4pq}{d^{2}}$). Previous Indian research documented substantial variation in waiting times across OPDs [16]. Because the study also examined exploratory associations, the calculated sample size was not based on a prespecified effect size for the analytic comparisons.

Because no directly comparable prevalence estimate was available for the defined outcome, p was set at 50% to obtain the minimum required sample size, with q set at 50% and the absolute precision (d) at 5%.

$n = \frac{4 \times 50 \times 50}{5^{2}} = 400 \text{ participants}$

Study variables and outcomes

The primary outcomes were the distributions of registration waiting time, pre-consultation waiting time, total OPD waiting time, and doctor consultation duration. Secondary outcomes included satisfaction with registration waiting, pre-consultation waiting, consultation duration, and the overall waiting experience, together with willingness to revisit the hospital.

The explanatory variables included age, gender, socioeconomic status, residence, OPD attended, visit type, arrival time, and the participant’s perceived main reason for waiting. Total waiting time was treated as the outcome when its associations with participant and visit characteristics were assessed and as the explanatory variable when its relationships with overall waiting-time satisfaction and willingness to revisit were examined. No effect modifiers were prespecified. The categorical and correlation analyses were unadjusted. For the multivariable analysis of overall waiting-time satisfaction, age group, gender, socioeconomic status, residence, OPD, visit type, and arrival time were included as covariates.

Data collection

A structured, pretested, bilingual English-Hindi questionnaire was used to collect demographic characteristics, department attended, visit type, arrival-time category, perceived main reason for waiting, approximate waiting and consultation-time categories, satisfaction with different stages of the OPD process, and willingness to revisit. Eligible participants were identified after registration while awaiting consultation. The questionnaire was administered immediately after the doctor consultation had been completed. Thus, the study sequence was arrival, registration, pre-consultation waiting, doctor consultation, and questionnaire administration. Participants were asked to retrospectively report their approximate registration waiting time, pre-consultation waiting time, doctor consultation duration, and total OPD waiting time by selecting one of the predefined ordered categories. Satisfaction was recorded on a five-point Likert scale, and willingness to revisit was recorded as yes, no, or not sure. The questionnaire was administered in English or Hindi according to each participant’s preference and is provided in the Appendices.

Operational definitions

Registration waiting time was defined as the interval from arrival at the common registration service point to completion of registration.

Pre-consultation waiting time was defined as the interval from completion of registration to the beginning of the doctor's consultation.

Doctor consultation duration was defined as the interval from the beginning to the completion of the consultation. This was considered a service-time measure rather than a waiting-time measure.

Total OPD waiting time was defined as the interval from the beginning of registration to the start of the doctor's consultation.

Visit type was classified as a first visit or a follow-up visit for the same health condition.

Registration waiting time, pre-consultation waiting time, and consultation duration were categorized as <15, 15-30, 31-60, and >60 minutes. Total waiting time was categorized as <1, one to two, two to three, and >3 hours.

Patient satisfaction was assessed using a five-point Likert scale ranging from very satisfied to very dissatisfied. For correlation analysis, satisfaction was coded from 1 (very satisfied) to 5 (very dissatisfied). Thus, higher scores represented poorer satisfaction.

Willingness to revisit was recorded as yes, no, or not sure.

Potential sources of bias

Convenience sampling may have introduced selection bias because recruitment was limited to patients available during routine outpatient activity. Standardized eligibility criteria, a structured bilingual questionnaire, predefined operational definitions, and identical categorical response options were used to improve consistency across participants and departments.

Waiting and consultation times, satisfaction, and willingness to revisit were self-reported and could have been affected by recall error, individual expectations, response tendencies, or social desirability. Broad ordered time categories may have reduced recall demands but also limited measurement precision. Patients receiving emergency, priority, or expedited services were excluded because their pathway differed from routine outpatient care. Nevertheless, residual selection, measurement, and response bias could not be eliminated.

Statistical analysis

Data were analyzed using IBM SPSS Statistics for Windows, version 20.0 (IBM Corp., Armonk, NY).

Categorical variables were summarized using frequencies and percentages. Because waiting and consultation times were recorded as ordered categories rather than exact continuous measurements, means and standard deviations were not calculated.

Pearson's chi-square test was used to examine associations between categorical variables. When small expected frequencies made the asymptotic Pearson p-value unsuitable, Monte Carlo significance estimates were used. Cramér's V was reported to describe the magnitude of categorical associations.

Spearman's rank correlation was used to assess ordered relationships between waiting or consultation-time categories and corresponding satisfaction scores. All tests were two-sided, with p < 0.05 considered statistically significant for the overall analyses. To account for specialty-specific operational constraints - such as rapid diagnostic triage in high-volume clinics versus minor procedures or detailed evaluations in surgical and subspecialty units - the OPD was included as a covariate alongside age group, gender, socioeconomic status, residence, visit type, and arrival-time category. A multivariable proportional-odds ordinal logistic regression model was fitted with overall waiting-time satisfaction as the ordinal dependent variable, coded from 1 (very satisfied) to 5 (very dissatisfied). Total OPD waiting time was entered as the principal explanatory variable, coded ordinally from 1 (<1 hour) to 4 (>3 hours). Adjusted odds ratios (aORs), 95% confidence intervals (CIs), and two-sided p-values were reported, where an aOR greater than 1 indicated higher odds of reporting worse satisfaction. Descriptive and bivariate analyses were conducted using IBM SPSS Statistics for Windows, version 20.0, while the multivariable ordinal model was fitted using Python 3.12 (Python Software Foundation, Wilmington, DE) with SciPy 1.17.0 (Travis Oliphant, Pearu Peterson, and Eric Jones, Rochester, MN).

For exploratory department-stratified analyses, the Benjamini-Hochberg procedure was used to control the false discovery rate. An adjusted q < 0.05 was considered statistically significant.

The convenience-sampling strategy was not incorporated through sampling weights or design-based variance estimation. All 400 participants had complete information for the variables included in the final analyses; therefore, complete-case analysis was used, and no imputation was required. No sensitivity analyses were performed.

The analysis was designed to report the associations actually examined without interpreting non-significant findings beyond what the data supported.

Ethical considerations

The study was approved by the Institutional Ethics Committee (IEC) of Rajendra Institute of Medical Sciences, Ranchi (IEC approval number 61; submission/reference number S/51/26; approval date: February 24, 2026). Permission was obtained from the Department of Hospital Administration. Written informed consent was obtained from all participants. Participation was voluntary, and confidentiality and anonymity were maintained. The observational procedures did not interfere with routine patient care.

## Results

Participant characteristics

A total of 400 participants were included in the analysis, with no missing observations in the 21 variables contained in the master chart. The sample comprised 193 (48.3%) male participants and 207 (51.8%) female participants. The largest age group was 40-49 years, comprising 126 (31.5%) participants. Graduates accounted for 153 (38.3%), the lower-middle socioeconomic group for 168 (42.0%), Hindus for 235 (58.8%), employed participants for 118 (29.5%), and urban residents for 157 (39.3%). New visits accounted for 233 (58.3%) participants, and 277 (69.3%) arrived before 11:00 AM. High patient load, delay in registration, and delayed doctor availability were each selected as the main perceived reason for waiting by 102 (25.5%) participants, while 94 (23.5%) selected poor queue management.

Participants were distributed across eight OPDs, with 45-55 participants represented in each department. The demographic and visit-related characteristics are summarized in Table 1. 

**Table 1:** Sociodemographic and visit-related characteristics of study participants (N = 400) Values are presented as n (%). Percentages may not total exactly 100% because of rounding.

Characteristic	Category	n (%)
Age group	20-29 years	90 (22.5)
	30-39 years	103 (25.8)
	40-49 years	126 (31.5)
	≥50 years	81 (20.2)
Gender	Male	193 (48.3)
	Female	207 (51.8)
Socioeconomic status	Upper	43 (10.8)
	Upper-middle	113 (28.2)
	Lower-middle	168 (42.0)
	Lower	76 (19.0)
Residence	Rural	124 (31.0)
	Semi-urban	119 (29.8)
	Urban	157 (39.3)
Visit type	New	233 (58.3)
	Follow-up	167 (41.8)
Arrival time	9:00-10:00 AM	162 (40.5)
	10:00-11:00 AM	115 (28.7)
	11:00 AM-1:00 PM	81 (20.2)
	After 2:00 PM	42 (10.5)
Outpatient department	Dermatology	55 (13.8)
	ENT	53 (13.2)
	Gynecology	47 (11.8)
	Medicine	46 (11.5)
	Neurology	55 (13.8)
	Ophthalmology	45 (11.3)
	Orthopedics	51 (12.8)
	Surgery	48 (12.0)
Education	Diploma	43 (10.8)
	Graduate	153 (38.3)
	Postgraduate	117 (29.3)
	Other	87 (21.8)
Religion	Hindu	235 (58.8)
	Muslim	91 (22.8)
	Christian	38 (9.5)
	Sikh	13 (3.3)
	Buddhist	6 (1.5)
	Jain	4 (1.0)
	Other	5 (1.3)
	Prefer not to say	8 (2.0)
Occupation	Student	41 (10.3)
	Homemaker	78 (19.5)
	Employed	118 (29.5)
	Self-employed	83 (20.8)
	Unemployed	45 (11.3)
	Retired	35 (8.8)
Perceived main reason for waiting	High patient load	102 (25.5)
	Delay in registration	102 (25.5)
	Doctor not available on time	102 (25.5)
	Poor queue management	94 (23.5)

Gender distribution differed significantly across departments (χ²(7) = 53.246, p < 0.001; Cramér's V = 0.365), largely because all 47 participants attending the Gynecology OPD were female. Gender was not significantly associated with age group, socioeconomic status, residence, visit type, or arrival time (all p > 0.05).

Waiting and consultation time

The distribution of the different components of the outpatient journey is presented in Table 2 and Figure 1. 

**Table 2:** Distribution of waiting and consultation times among study participants (N = 400)

Time variable	Category	n	%
Registration waiting	<15 minutes	101	25.3
	15-30 minutes	99	24.8
	31-60 minutes	96	24.0
	>60 minutes	104	26.0
Pre-consultation waiting	<15 minutes	51	12.8
	15-30 minutes	129	32.3
	31-60 minutes	101	25.3
	>60 minutes	119	29.8
Doctor consultation duration	<15 minutes	151	37.8
	15-30 minutes	172	43.0
	31-60 minutes	63	15.8
	>60 minutes	14	3.5
Total outpatient waiting	<1 hour	47	11.8
	1-2 hours	62	15.5
	2-3 hours	58	14.5
	>3 hours	233	58.3

**Figure 1:**
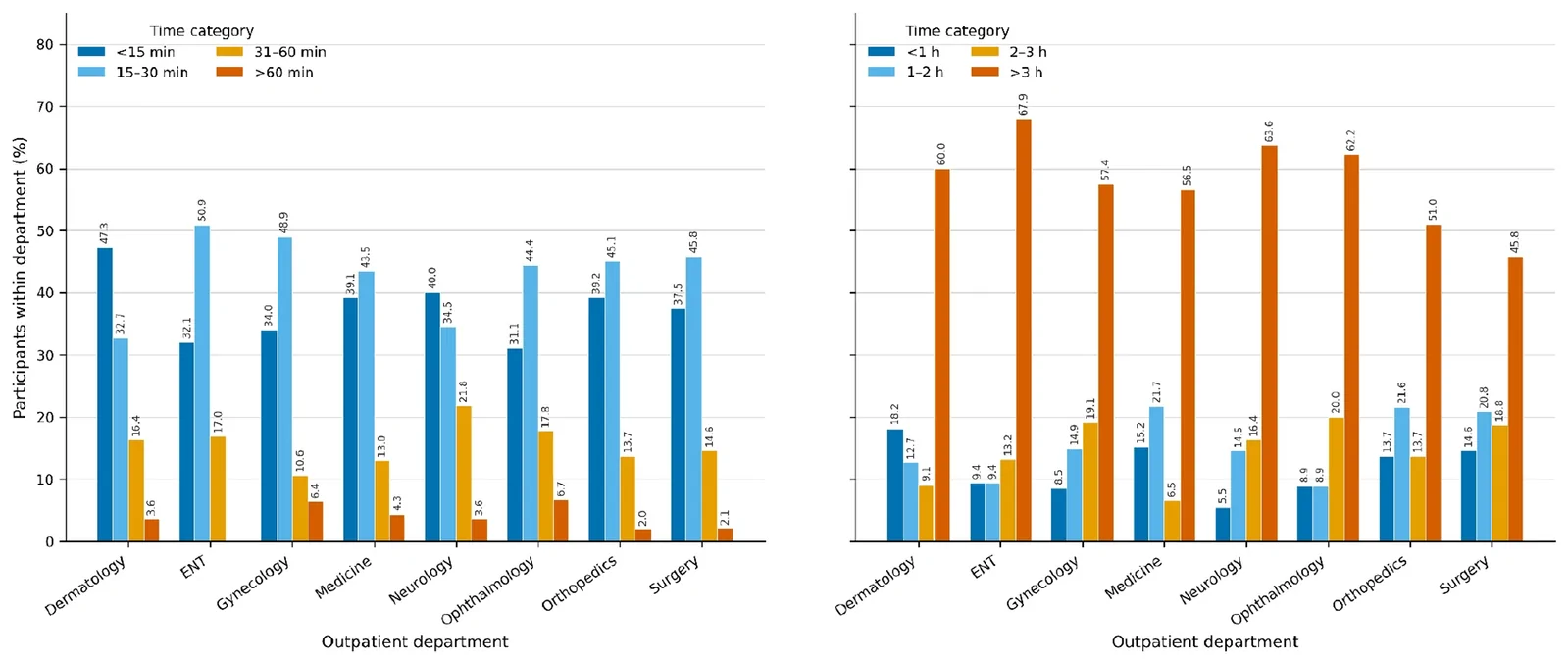
Department-wise distributions of doctor consultation duration and total outpatient waiting time The left panel presents doctor consultation-duration categories, and the right panel presents total outpatient waiting-time categories across the eight departments. Values above the bars represent percentages calculated within each department. Departmental differences were not statistically significant for consultation duration (Monte Carlo p = 0.885) or total waiting time (p = 0.493).

At registration, 101 (25.2%) participants waited less than 15 minutes, 99 (24.8%) waited 15-30 minutes, 96 (24.0%) waited 31-60 minutes, and 104 (26.0%; 95% CI: 21.9%-30.5%) waited more than 60 minutes.

After registration, 51 (12.8%) participants waited less than 15 minutes before consultation, 129 (32.2%) waited 15-30 minutes, 101 (25.2%) waited 31-60 minutes, and 119 (29.8%; 95% CI: 25.5%-34.4%) waited more than 60 minutes.

Doctor consultation duration was 30 minutes or less for 323 (80.8%; 95% CI: 76.6%-84.3%) participants, including 151 (37.8%) whose consultations lasted less than 15 minutes and 172 (43.0%) whose consultations lasted 15-30 minutes. Only 14 (3.5%) consultations lasted more than 60 minutes.

Total OPD waiting time was less than one hour for 47 (11.8%) participants, one to two hours for 62 (15.5%), two to three hours for 58 (14.5%), and more than three hours for 233 (58.3%; 95% CI: 53.3%-63.1%).

Department-wise waiting patterns

The distribution of waiting and consultation time according to the department is presented in Table 3. 

**Table 3:** Waiting and consultation-time distributions according to outpatient department (N = 400) Category definitions: for registration waiting, pre-consultation waiting, and consultation duration, Categories 1-4 represent <15, 15-30, 31-60, and >60 minutes, respectively. For total waiting, Categories 1-4 represent <1, one to two, two to three, and >3 hours, respectively. Values are presented as n (row %), with percentages calculated within each department. Percentages may not total exactly 100% because of rounding. Registration waiting: Pearson χ²(21) = 16.335, p = 0.751; Cramér's V = 0.117. Pre-consultation waiting: Pearson χ²(21) = 29.838, p = 0.095; Cramér's V = 0.158. Consultation duration: Pearson χ²(21) = 13.806, Monte Carlo p = 0.885; Cramér's V = 0.107. Total waiting: Pearson χ²(21) = 20.445, p = 0.493; Cramér's V = 0.131.

Time measure	Department	Category 1 n (%)	Category 2 n (%)	Category 3 n (%)	Category 4 n (%)	Total, n
Registration waiting	Dermatology	14 (25.5)	13 (23.6)	11 (20.0)	17 (30.9)	55
	ENT	11 (20.8)	14 (26.4)	16 (30.2)	12 (22.6)	53
	Gynecology	11 (23.4)	12 (25.5)	12 (25.5)	12 (25.5)	47
	Medicine	16 (34.8)	8 (17.4)	9 (19.6)	13 (28.3)	46
	Neurology	11 (20.0)	11 (20.0)	13 (23.6)	20 (36.4)	55
	Ophthalmology	7 (15.6)	13 (28.9)	12 (26.7)	13 (28.9)	45
	Orthopedics	16 (31.4)	14 (27.5)	12 (23.5)	9 (17.6)	51
	Surgery	15 (31.2)	14 (29.2)	11 (22.9)	8 (16.7)	48
	Total	101 (25.2)	99 (24.8)	96 (24.0)	104 (26.0)	400
Pre-consultation waiting	Dermatology	12 (21.8)	12 (21.8)	18 (32.7)	13 (23.6)	55
	ENT	7 (13.2)	18 (34.0)	12 (22.6)	16 (30.2)	53
	Gynecology	3 (6.4)	18 (38.3)	12 (25.5)	14 (29.8)	47
	Medicine	4 (8.7)	16 (34.8)	11 (23.9)	15 (32.6)	46
	Neurology	6 (10.9)	15 (27.3)	9 (16.4)	25 (45.5)	55
	Ophthalmology	4 (8.9)	10 (22.2)	16 (35.6)	15 (33.3)	45
	Orthopedics	7 (13.7)	18 (35.3)	11 (21.6)	15 (29.4)	51
	Surgery	8 (16.7)	22 (45.8)	12 (25.0)	6 (12.5)	48
	Total	51 (12.8)	129 (32.2)	101 (25.2)	119 (29.8)	400
Consultation duration	Dermatology	26 (47.3)	18 (32.7)	9 (16.4)	2 (3.6)	55
	ENT	17 (32.1)	27 (50.9)	9 (17.0)	0 (0.0)	53
	Gynecology	16 (34.0)	23 (48.9)	5 (10.6)	3 (6.4)	47
	Medicine	18 (39.1)	20 (43.5)	6 (13.0)	2 (4.3)	46
	Neurology	22 (40.0)	19 (34.5)	12 (21.8)	2 (3.6)	55
	Ophthalmology	14 (31.1)	20 (44.4)	8 (17.8)	3 (6.7)	45
	Orthopedics	20 (39.2)	23 (45.1)	7 (13.7)	1 (2.0)	51
	Surgery	18 (37.5)	22 (45.8)	7 (14.6)	1 (2.1)	48
	Total	151 (37.8)	172 (43.0)	63 (15.8)	14 (3.5)	400
Total waiting	Dermatology	10 (18.2)	7 (12.7)	5 (9.1)	33 (60.0)	55
	ENT	5 (9.4)	5 (9.4)	7 (13.2)	36 (67.9)	53
	Gynecology	4 (8.5)	7 (14.9)	9 (19.1)	27 (57.4)	47
	Medicine	7 (15.2)	10 (21.7)	3 (6.5)	26 (56.5)	46
	Neurology	3 (5.5)	8 (14.5)	9 (16.4)	35 (63.6)	55
	Ophthalmology	4 (8.9)	4 (8.9)	9 (20.0)	28 (62.2)	45
	Orthopedics	7 (13.7)	11 (21.6)	7 (13.7)	26 (51.0)	51
	Surgery	7 (14.6)	10 (20.8)	9 (18.8)	22 (45.8)	48
	Total	47 (11.8)	62 (15.5)	58 (14.5)	233 (58.3)	400

Registration waiting time did not differ significantly across departments (χ²(21) = 16.335, p = 0.751; Cramér's V = 0.117). The proportion waiting more than 60 minutes ranged from 16.7% in Surgery to 36.4% in Neurology. 

Pre-consultation waiting time also did not differ significantly across departments (χ²(21) = 29.838, p = 0.095; Cramér's V = 0.158). The proportion waiting more than 60 minutes ranged from 12.5% in Surgery to 45.5% in Neurology.

Doctor consultation duration did not differ significantly across departments (χ²(21) = 13.806; Monte Carlo p = 0.885; Cramér's V = 0.107). Consultations lasting 30 minutes or less accounted for most encounters in each department.

Total OPD waiting time also did not differ significantly by department (χ²(21) = 20.445, p = 0.493; Cramér's V = 0.131). Waiting for more than three hours was the most frequent category in every department, ranging from 45.8% in Surgery to 67.9% in ENT.

Factors associated with total waiting time

Total waiting time was significantly associated with visit type (χ²(3) = 51.096, p < 0.001; Cramér’s V = 0.357) and arrival time (χ²(9) = 179.485, p < 0.001; Cramér’s V = 0.387). Other demographic variables examined in the overall analysis were not significantly associated with total waiting time. These findings indicate that characteristics related to how and when patients entered the OPD pathway were more clearly associated with total waiting time than the demographic variables examined in this study.

Waiting time and satisfaction

The relationships between time spent at different stages of the OPD pathway and corresponding satisfaction were statistically significant and strong.

Registration waiting time showed a strong positive correlation with poorer registration satisfaction (Spearman's ρ = 0.879, p < 0.001). Similarly, longer pre-consultation waiting was strongly associated with poorer satisfaction with the pre-consultation waiting period (ρ = 0.871, p < 0.001).

Total OPD waiting time was also strongly associated with poorer satisfaction with the overall waiting experience (ρ = 0.816, p < 0.001).

In contrast, doctor consultation duration showed an inverse association with consultation dissatisfaction (ρ = -0.668, p < 0.001), indicating that participants with longer consultations tended to report better consultation-related satisfaction.

All 32 department-specific correlations remained statistically significant after Benjamini-Hochberg correction for multiple testing (all adjusted q < 0.001) (Table 4). 

**Table 4:** Spearman correlations between waiting or consultation time and the corresponding satisfaction measure (N = 400) Satisfaction was coded from 1 (very satisfied) to 5 (very dissatisfied). Accordingly, positive coefficients indicate poorer satisfaction as waiting time increases, while the negative coefficient for consultation duration indicates better satisfaction with longer consultations.

Variable	Satisfaction outcome	Coefficient	p-value
Registration waiting time	Registration satisfaction	0.879	<0.001
Pre-consultation waiting time	Pre-consultation satisfaction	0.871	<0.001
Doctor consultation duration	Consultation satisfaction	-0.668	<0.001
Total outpatient department waiting time	Overall waiting-time satisfaction	0.816	<0.001

Multivariable analysis of overall satisfaction

A multivariable ordinal logistic regression model was fitted to examine factors associated with overall patient dissatisfaction (Table 5). After adjustment for age group, gender, socioeconomic status, residence, OPD, visit type, and arrival time, each one-category increase in total waiting time was associated with higher odds of belonging to a worse satisfaction category (aOR = 22.75, 95% CI: 13.68-37.83; p < 0.001). None of the other included covariates reached statistical significance. The overall model was statistically significant (likelihood-ratio χ²(21) = 470.93; p < 0.001) (Table 5). 

**Table 5:** Multivariable ordinal logistic regression of factors associated with poorer overall waiting-time satisfaction (N = 400) aOR = adjusted odds ratio; CI = confidence interval aORs were calculated using multivariable ordinal logistic regression. An aOR >1 indicates greater odds of poorer waiting-time satisfaction. Reference categories are shown in the table. Bold value indicates p < 0.05.

Predictor	aOR	95% CI	p-value
Total waiting time, per one-category increase	22.75	13.68-37.83	<0.001
Age 30-39 vs. 20-29 years	0.77	0.43-1.40	0.395
Age 40-49 vs. 20-29 years	0.92	0.52-1.61	0.762
Age ≥50 vs. 20-29 years	0.73	0.39-1.36	0.317
Female vs. male	1	0.51-1.21	0.276
Upper-middle vs. upper socioeconomic status	0.6	0.29-1.25	0.171
Lower-middle vs. upper socioeconomic status	1.12	0.56-2.24	0.759
Lower vs. upper socioeconomic status	0.93	0.43-2.01	0.845
Semi-urban vs. rural	0.62	0.37-1.04	0.071
Urban vs. rural	0.7	0.43-1.14	0.15
ENT vs. Dermatology	1.21	0.55-2.68	0.633
Gynecology vs. Dermatology	1.2	0.52-2.75	0.67
Medicine vs. Dermatology	1.26	0.56-2.83	0.573
Neurology vs. Dermatology	1.04	0.47-2.30	0.925
Ophthalmology vs. Dermatology	1.09	0.48-2.47	0.843
Orthopedics vs. Dermatology	0.91	0.41-2.02	0.816
Surgery vs. Dermatology	1.06	0.47-2.40	0.885
Follow-up vs. new visit	1.01	0.63-1.64	0.958
Arrival at 10-11 AM vs. 9-10 AM	1.26	0.72-2.23	0.415
Arrival at 11 AM-1 PM vs. 9-10 AM	1.2	0.59-2.45	0.614
Arrival after 2 PM vs. 9-10 AM	2.33	0.99-5.51	0.053

Willingness to revisit

The relationship between total OPD waiting time and willingness to revisit is shown in Table 6 and Figure 2. 

**Table 6:** Association between total outpatient department waiting time and willingness to revisit the hospital (N = 400) Pearson χ²(6) = 249.166, p < 0.001; Cramér's V = 0.558. Percentages are calculated within each total waiting time category.

Total waiting time (in hour)	Yes, n (%)	No, n (%)	Not sure, n (%)	Total
<1 hour	37 (78.7)	2 (4.3)	8 (17.0)	47
1-2 hours	44 (71.0)	7 (11.3)	11 (17.7)	62
2-3 hours	13 (22.4)	22 (37.9)	23 (39.7)	58
>3 hours	0 (0.0)	174 (74.7)	59 (25.3)	233
Total	94 (23.5)	205 (51.2)	101 (25.2)	400

**Figure 2:**
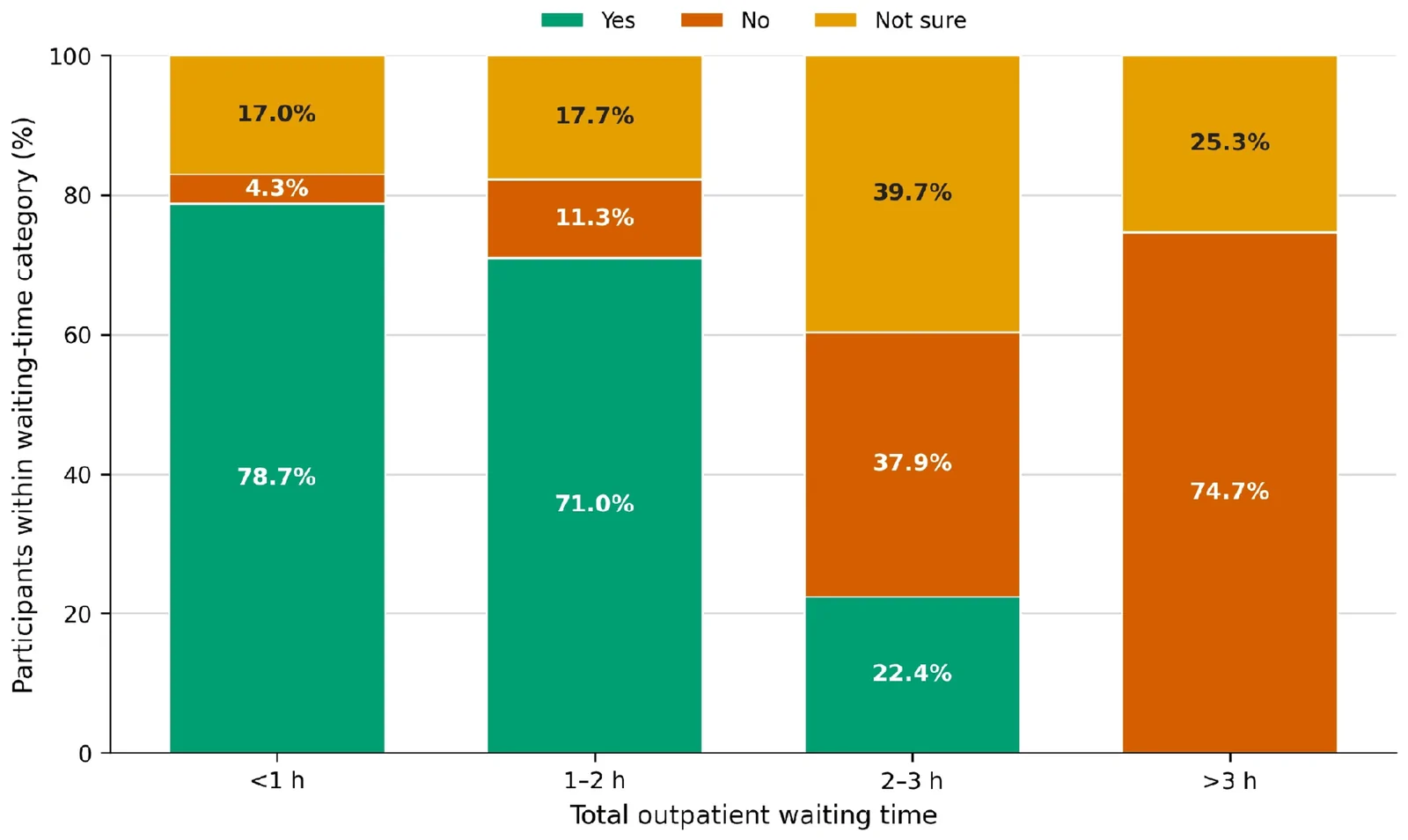
Willingness to revisit the hospital according to total outpatient waiting time Each bar represents all participants in the corresponding total waiting time category. Segment labels show percentages within that category. Willingness to revisit declined as total waiting time increased; the association was statistically significant (χ²(6) = 249.166, p < 0.001; Cramér’s V = 0.558).

Among participants who waited less than one hour, 37 (78.7%) reported that they would revisit the hospital. This proportion decreased to 44 (71.0%) among those waiting one to two hours and to 13 (22.4%) among those waiting two to three hours.

Among the 233 participants who waited more than three hours, none reported that they would revisit. In this group, 174 (74.7%) stated that they would not return, while 59 (25.3%) were unsure.

The association between total waiting time and willingness to revisit was statistically significant and substantial in magnitude (χ²(6) = 249.166, p < 0.001; Cramér's V = 0.558).

## Discussion

Principal findings

This study examined the outpatient journey as a sequence of waiting and consultation stages among 400 adults attending a high-volume tertiary care teaching hospital in Jharkhand. The most notable finding was the considerable difference between time spent waiting and time spent in direct clinical consultation. More than half of the participants (58.3%) waited more than three hours from the beginning of registration to the start of consultation, whereas 80.8% completed their consultation within 30 minutes.

Registration waiting exceeded 60 minutes for 26.0% of participants, and 29.8% waited more than 60 minutes after registration before seeing the doctor. Although waiting and consultation-time distributions did not differ significantly across departments, total waiting was associated with visit type and arrival time. Longer waiting at registration, before consultation, and across the overall OPD journey was consistently associated with poorer satisfaction, whereas longer consultation duration was associated with better consultation-related satisfaction. Total waiting was also strongly associated with willingness to revisit.

Comparison with previous studies

The predominance of waiting over direct clinical contact is consistent with findings from several outpatient settings. Studies from Nigeria [1], Iran [2], and Ethiopia [3] have described substantial outpatient waiting and have identified patient volume, service organization, and arrival patterns as potential contributors. In India, Langstieh et al. reported a mean waiting time of 110.86 minutes compared with a mean consultation duration of only 5.34 minutes in a neurology OPD [4]. Other Indian studies from Gujarat [5], Maharashtra [6], northern India [7], and Uttarakhand [8] have similarly reported substantial outpatient waiting time in relation to consultation duration and patient satisfaction.

Direct numerical comparisons should, however, be made cautiously because studies differ in patient populations, service settings, definitions of waiting time, and measurement methods. The present study used ordered time categories rather than exact continuous measurements and therefore did not calculate mean or median waiting times.

Department-wise waiting patterns

The absence of statistically significant department-level differences suggests that prolonged waiting was not confined to a particular specialty during the study period. This does not mean that the underlying workflows were identical. Departmental sample sizes were relatively modest, and the departments were represented through routine attendance rather than equal prospective allocation.

Nevertheless, waiting for more than three hours was the most frequent total waiting time category across all departments. These same four categories were used consistently for pre-consultation waiting time, as reported in Table 2 and Table 3, and for the corresponding correlation with satisfaction in Table 4. This consistent pattern across departments may indicate that delays arise partly from processes shared across departments, such as registration congestion, common arrival peaks, patient navigation, and cumulative queues. Previous studies have similarly identified patient volume, process design, and local organizational factors as important contributors to outpatient waiting times [7,16-21]. Although the overall department comparison was not statistically significant, potentially important descriptive variations were observed. Neurology had the highest proportion of participants with pre-consultation waiting exceeding 60 minutes (45.5%). This may reflect detailed neurological assessment, review of previous investigations, prioritization according to clinical complexity, or dependence on specialized diagnostic services. ENT had the highest proportion with total waiting exceeding three hours (67.9%). Possible contributors include minor procedures, endoscopic or audiological assessment, shared examination rooms or equipment, and variation in clinician availability or consultation start times. These explanations remain hypotheses because specialty-specific workflow variables, diagnostic procedures, staffing levels, room availability, and physician punctuality were not directly measured.

Factors associated with total waiting time

Visit type and arrival time were the main operational factors associated with total waiting time in the present study. New patients may require additional steps, such as record creation, documentation, registration assistance, or navigation through an unfamiliar hospital system. Similarly, patients arriving later may encounter queues that have accumulated earlier in the day. Importantly, departmental differences were not statistically significant for consultation duration (Monte Carlo p = 0.885) or total OPD waiting time (p = 0.493), suggesting that these operational factors may have a greater influence on waiting time than the specific department attended.

Previous studies have reported associations between outpatient waiting and patient characteristics, arrival patterns, and service organization [2,3,18-20]. The present findings reinforce the importance of examining operational factors rather than assuming that waiting is primarily determined by demographic characteristics.

Total OPD waiting time was significantly associated with visit type and arrival time, suggesting that characteristics related to when and how patients entered the outpatient pathway were more relevant than the demographic characteristics examined. No statistically significant associations were observed with age, gender, socioeconomic status, or residence; however, these null findings should be interpreted cautiously because some subgroup sizes were limited.

Patient satisfaction and consultation duration

The strong association between longer waiting and poorer satisfaction is consistent with previous research. Studies from Gujarat and Maharashtra have reported relationships between waiting time, service time, and outpatient satisfaction [5,6]. Similar relationships between waiting time and satisfaction have been reported in other outpatient settings [9-12], while a recent study from Uttarakhand also identified waiting-related services as an important component of outpatient satisfaction [22].

An important feature of the present study is that waiting was examined at several stages of the same patient journey. Registration waiting, pre-consultation waiting, and total waiting were each strongly associated with corresponding satisfaction measures. Notably, ENT had the highest proportion of participants with total waiting exceeding three hours (67.9%), and Neurology had the highest proportion with pre-consultation waiting exceeding 60 minutes (45.5%), suggesting these two departments may benefit most from targeted workflow review. This suggests that patients may experience outpatient care as a sequence of delays rather than as a single waiting period.

Consultation duration showed a different pattern from waiting times. While total OPD waiting time was strongly associated with willingness to revisit-and not merely with overall waiting-time satisfaction-consultation duration was not associated with willingness to revisit. Instead, longer consultations were consistently associated with higher satisfaction with consultation duration across all participating departments, even after adjusting for multiple comparisons. This finding aligns with research emphasizing the need to distinguish waiting time from service time [11]. Longer consultations likely enhance satisfaction with consultation duration by allowing thorough explanations, greater perceived attentiveness, and more opportunity to ask questions, whereas shorter encounters may feel rushed regardless of clinical adequacy. Consequently, while total OPD delays drive broader patient dissatisfaction and future care-seeking decisions, consultation length specifically shapes satisfaction with the clinical encounter itself.

The implication is important for quality improvement. Reducing outpatient waiting should not simply mean moving patients through the system faster at the expense of clinical interaction. Efforts should instead focus on removing avoidable administrative and queue delays while preserving sufficient time for examination, explanation, communication, and patient questions.

Willingness to revisit

Willingness to revisit declined markedly as total waiting time increased. Among participants waiting less than one hour, 78.7% reported that they would revisit the hospital, compared with none among those waiting more than three hours. The association was statistically significant and substantial.

Although this finding has practical importance for patient experience and service continuity, willingness to revisit represents an intention rather than observed future utilization. It may also be influenced by factors other than waiting, including perceived quality of care, affordability, travel distance, referral requirements, and availability of alternative hospitals.

These observed relationships may be influenced by unmeasured factors such as perceived quality of care, communication, affordability, travel distance, and availability of alternative services. Nevertheless, the strong gradient observed across waiting categories supports further prospective investigation to better understand patient flow dynamics and service utilization.

Implications

The next step should be a prospective workflow audit using electronic or directly observed timestamps for arrival, registration initiation and completion, entry into the departmental queue, consultation initiation, and consultation completion. These measurements should be linked with hourly patient volume, staffing, clinician start time, queue length, room and equipment availability, diagnostic procedures, and service interruptions. Department-specific process mapping should initially prioritize Neurology and ENT because of their comparatively high proportions of prolonged waiting.

These detailed process data would distinguish active service delivery time - such as in-clinic diagnostic sorting or minor procedures - from avoidable administrative delays across registration and pre-consultation queues. Recognizing these distinct operational bottlenecks supports a system-wide approach to optimizing the outpatient pathway rather than relying on isolated departmental adjustments.

Based on the audit findings, locally appropriate interventions such as electronic preregistration, token-based queue management, clearer wayfinding, assistance for new patients, staggered appointments, and staffing aligned with peak arrival periods should be piloted. A recent study protocol similarly highlighted direct observation of outpatient flow and optimized resource utilization as key strategies for delay reduction [23]. More advanced queueing theory and integral patient scheduling models may also prove beneficial where reliable operational data exist [24,25]. The effect of these interventions should be rigorously evaluated using before-and-after or controlled prospective designs that assess waiting time, patient satisfaction, observed revisit behavior, and consultation duration to ensure that reductions in delay do not compromise clinical interaction.

Strengths and limitations

This study assessed four sequential components of the outpatient journey: registration waiting, pre-consultation waiting, consultation duration, and total waiting. It also examined stage-specific satisfaction and willingness to revisit among patients from eight clinical departments represented during routine data collection.

The use of ordered categories allowed consistent measurement across the outpatient pathway, and effect sizes were reported alongside statistical significance. False discovery rate adjustment was used for exploratory department-specific analyses.

Several limitations should be considered. Convenience sampling may have introduced selection bias because patients who were present and willing to participate may have differed systematically from other outpatient attendees. The absence of a pre-enrollment screening log prevented calculation of a response or participation rate; thus, self-selection bias cannot be excluded. Patients with sufficient time to complete the questionnaire or those experiencing less acute frustration may have been more likely to participate, and exclusion of patients unable to complete the routine pathway may have underrepresented those with interrupted or atypical visits. Department-specific sample sizes were modest, which limited statistical power to detect subtle departmental variations.

Furthermore, grouping eight diverse clinical departments under uniform time categories overlooks specialty-specific operational constraints-such as rapid diagnostic sorting versus time-intensive minor procedures or detailed subspecialty evaluations. Waiting and consultation times were reported by participants in broad ordered categories rather than measured as exact timestamps, exposing the data to recall and classification error, limiting measurement precision, and preventing calculation of exact central tendencies or operational thresholds. Unmeasured operational factors - including daily patient volume, staffing levels, clinician punctuality, queue length, service interruptions, and day-of-week effects - may have introduced residual confounding. Self-reported satisfaction and willingness to revisit were subject to response bias driven by individual expectations or social desirability. The direction and magnitude of these potential biases could not be determined. Finally, the cross-sectional design establishes associations but cannot establish causality.

The findings are most applicable to high-volume tertiary care teaching hospitals with outpatient pathways and patient volumes similar to those of the study institution. Their applicability to private hospitals, secondary-care facilities, appointment-based clinics, and hospitals with substantially different registration or queue-management systems may be limited. Despite these limitations, the consistent relationship between prolonged waiting, poorer satisfaction, and reduced willingness to revisit provides a basis for further pathway-specific quality-improvement work.

## Conclusions

This cross-sectional study demonstrates that prolonged outpatient waiting was highly prevalent, with 58.3% of participants waiting over three hours from registration to consultation. Longer total waiting time was an independent predictor of poorer patient satisfaction and reduced willingness to revisit, whereas longer consultation duration correlated with higher consultation-specific satisfaction. Although total waiting times were significantly influenced by visit type and arrival time rather than departmental differences, these findings highlight the necessity of streamlining early administrative and pre-consultation queues-particularly during peak morning hours and for new patients-without compromising clinical interaction time.

## Appendices

**Table 7:** Questionnaire used in the study

Patient waiting time questionnaire परिशिष्ट A रोगियों के प्रतीक्षा समय की प्रश्नावली	
Study title: An Observational Study on Patients’ Waiting Time in the Outpatient Department of a Tertiary Care Teaching Hospital in Jharkhand. अध्ययन शीर्षक: झारखंड के एक तृतीयक देखभाल शिक्षण अस्पताल के बाह्य रोगी विभाग में रोगियों के प्रतीक्षा समयपर एक प्रेक्षणात्मक अध्ययन	
Participant information and consent प्रतिभागी की जानकारी और सहमति	
Participation: Your participation is voluntary. You may withdraw at any time without consequences. The survey takes about 5 minutes. भागीदारी: आपकी सहभागिता स्वैच्छिक है। आप किसी भी समय बिना किसी प्रतिकूल परिणाम के अध्ययन से हट सकते हैं। सर्वेक्षणमें लगभग 5 मिनट लगेंगे।	
Confidentiality: Your responses and identifying information will be kept confidential and used only for research purposes. गोपनीयता: आपके उत्तर और पहचान संबंधी जानकारी गोपनीय रखी जाएगी और केवल शोध के उद्देश्य से उपयोग की जाएगी।	
Risks and benefits: This study involves no foreseeable risk. Your responses may help identify factors affecting OPD waiting time and contribute to improving patient care. जोखिम और लाभ: इस अध्ययन में किसी पूर्वानुमानित जोखिम की संभावना नहीं है। आपकेउत्तर ओपीडी में प्रतीक्षा समय को प्रभावित करने वाले कारकों की पहचान करने और रोगी देखभाल में सुधार करने में सहायक हो सकते हैं।	
Consent: By signing below, you confirm that you are an outpatient aged 20 years or older, that you have read or had the study information explained to you, that you understand the purpose of the study, and that you voluntarily agree to participate. सहमति: नीचे हस्ताक्षर करके आप पुष्टि करते हैं कि आप 20 वर्ष या उससे अधिक आयु के बाह्य रोगी हैं, आपने अध्ययन से संबंधितजानकारी पढ़ी है अथवा आपको समझाई गई है, आप अध्ययन के उद्देश्य को समझते हैं और स्वेच्छा से इसमें भाग लेने के लिए सहमत हैं।	
Signature of participant प्रतिभागी के हस्ताक्षर	
Instructions: Select one response for each question unless a written response is requested. / निर्देश: जहाँ लिखित उत्तर नहीं माँगागया है, वहाँ प्रत्येक प्रश्न के लिए एक विकल्प चुनें।	
Section A Demographic details अनुभाग A जनसांख्यिकीय विवरण	
1. Participant ID प्रतिभागी आईडी	
2. Name नाम	
3. Mobile number मोबाइल नंबर	
4. Email ID. ईमेल आईडी	
5. Age group / आयु समूह	☐ 20-29 years / वर्ष ☐ 30-39 years / वर्ष ☐ 40-49 years / वर्ष ☐ 50 years and above / 50 वर्ष एवं उससे अधिक
6. Gender / लिंग	☐ Male / पुरुष ☐ Female / महिला
7. Education / शैक्षणिक योग्यता	☐ Diploma / डिप्लोमा ☐ Graduate / स्नातक ☐Postgraduate / स्नातकोत्तर ☐ Other / अन्य
8. Socioeconomic status / सामाजिक आर्थिक स्थिति	☐ Upper class / उच्च वर्ग ☐ Upper middle class / उच्चमध्यम वर्ग ☐ Lower middle class / निम्न मध्यम वर्ग ☐Lower class / निम्न वर्ग
9. Religion / धर्म	☐ Hindu / हिन्दू ☐ Muslim / मुस्लिम ☐ Christian / ईसाई☐ Sikh / सिख ☐ Buddhist / बौद्ध ☐ Jain / जैन ☐ Other please specify / अन्य कृपया उल्लेख करें ☐ Prefer not to say / बताना नहीं चाहते
10. Occupation / व्यवसाय	☐ Student / छात्र या छात्रा ☐ Homemaker / गृहिणी यागृहस्थी संभालने वाले ☐ Employed / नौकरीपेशा ☐ Self-employed / स्वरोजगार ☐ Unemployed / बेरोजगार ☐Retired / सेवानिवृत्त
11. Place of residence / निवास स्थान	☐ Rural / ग्रामीण ☐ Semi-urban / अर्धशहरी ☐ Urban / शहरी
Section B OPD visit characteristics अनुभाग B ओपीडी में आने से संबंधित विवरण	
1. Is this your first visit to this OPD for the current health problem, or are you visiting for a follow-up consultation? क्या आप इस स्वास्थ्यसमस्या के लिए पहली बार इस ओपीडी में आए हैं या पुनः परामर्श के लिए आएहैं?	☐ New patient / नया रोगी ☐ Follow-up patient / पुनःपरामर्श के लिए आए रोगी
2. Please mention the department in which you are receiving consultation today. कृपया उस विभाग का नाम बताइए जहाँ आज आपपरामर्श ले रहे हैं।	
3. At approximately what time did you arrive at the OPD today? आजआप लगभग किस समय ओपीडी में पहुँचे थे?	☐ 9-10 AM / सुबह 9-10 बजे ☐ 10-11 AM / सुबह 10-11 बजे ☐ 11 AM-1 PM / सुबह 11 बजे से दोपहर 1 बजे तक ☐After 2 PM / दोपहर 2 बजे के बाद
Section C Waiting time assessment अनुभाग C प्रतीक्षा समय का आकलन	
4. Approximately how long did you wait at the registration counter before your registration was completed? पंजीकरण काउंटर पर पंजीकरणपूरा होने तक आपको लगभग कितनी देर प्रतीक्षा करनी पड़ी?	☐ <15 minutes / 15 मिनट से कम ☐ 15-30 minutes / 15-30 मिनट ☐ 31-60 minutes / 31-60 मिनट ☐ >60 minutes / 60 मिनट से अधिक
5. After completing your registration, approximately how long did you wait before you were called for consultation by the doctor? पंजीकरण पूरा होने के बाद डॉक्टर द्वारा परामर्श के लिए बुलाए जाने तक आपकोलगभग कितनी देर प्रतीक्षा करनी पड़ी?	☐ <15 minutes / 15 मिनट से कम ☐ 15-30 minutes / 15-30 मिनट ☐ 31-60 minutes / 31-60 मिनट ☐ >60 minutes / 60 मिनट से अधिक
6. Approximately how much time did the doctor spend in providing your consultation during today’s OPD visit? आज की ओपीडी यात्रा केदौरान डॉक्टर ने आपके परामर्श में लगभग कितना समय दिया?	☐ <15 minutes / 15 मिनट से कम ☐ 15-30 minutes / 15-30 मिनट ☐ 31-60 minutes / 31-60 मिनट ☐ >60 minutes / 60 मिनट से अधिक
7. From the beginning of registration until the beginning of your consultation with the doctor, what was your total waiting time in the OPD? पंजीकरण शुरू होने से लेकर डॉक्टर के साथ परामर्श शुरू होने तकओपीडी में आपका कुल प्रतीक्षा समय लगभग कितना था?	☐ <1 hour / 1 घंटे से कम ☐ 1-2 hours / 1-2 घंटे ☐ 2-3 hours / 2-3 घंटे ☐ >3 hours / 3 घंटे से अधिक
Section D Perceived reasons for waiting time अनुभाग D रोगी की राय में प्रतीक्षा समय के कारण	
8. According to your experience during today’s OPD visit, what do you think was the main reason for the waiting time you experienced? आज की ओपीडी यात्रा के अपने अनुभव के आधार पर, आपके अनुसार प्रतीक्षासमय का मुख्य कारण क्या था?	☐ High patient load / रोगियों की अधिक संख्या ☐ Delay in registration / पंजीकरण में देरी ☐ Doctor not available on time / डॉक्टर का समय पर उपलब्ध न होना ☐ Poor queue management / कतार प्रबंधन की कमी
Section E Patient satisfaction related to waiting time अनुभाग E प्रतीक्षा समय से संबंधित रोगी संतुष्टि	
9. Overall, how satisfied are you with the waiting time you experienced during today’s OPD visit? कुल मिलाकर, आज की ओपीडीयात्रा के दौरान हुई प्रतीक्षा के समय से आप कितने संतुष्ट हैं?	☐ 1 Very satisfied / बहुत संतुष्ट ☐ 2 Satisfied / संतुष्ट ☐3 Neutral / न संतुष्ट न असंतुष्ट ☐ 4 Dissatisfied / असंतुष्ट☐ 5 Very dissatisfied / बहुत असंतुष्ट
10. How satisfied are you with the time you had to wait at the registration counter before your registration was completed? पंजीकरण काउंटर पर पंजीकरण पूरा होने तक हुई प्रतीक्षा के समय से आपकितने संतुष्ट हैं?	☐ 1 Very satisfied / बहुत संतुष्ट ☐ 2 Satisfied / संतुष्ट ☐3 Neutral / न संतुष्ट न असंतुष्ट ☐ 4 Dissatisfied / असंतुष्ट☐ 5 Very dissatisfied / बहुत असंतुष्ट
11. How satisfied are you with the waiting time before your consultation with the doctor? डॉक्टर के साथ परामर्श शुरू होने से पहले हुईप्रतीक्षा के समय से आप कितने संतुष्ट हैं?	☐ 1 Very satisfied / बहुत संतुष्ट ☐ 2 Satisfied / संतुष्ट ☐3 Neutral / न संतुष्ट न असंतुष्ट ☐ 4 Dissatisfied / असंतुष्ट☐ 5 Very dissatisfied / बहुत असंतुष्ट
12. How satisfied are you with the amount of time the doctor spent during your consultation? डॉक्टर द्वारा आपके परामर्श में दिए गए समय सेआप कितने संतुष्ट हैं?	☐ 1 Very satisfied / बहुत संतुष्ट ☐ 2 Satisfied / संतुष्ट ☐3 Neutral / न संतुष्ट न असंतुष्ट ☐ 4 Dissatisfied / असंतुष्ट☐ 5 Very dissatisfied / बहुत असंतुष्ट
Section F Overall experience अनुभाग F समग्र अनुभव	
13. Would you prefer to visit this hospital again in the future for OPD services if required? यदि भविष्य में आवश्यकता पड़े, तो क्या आप इसअस्पताल की ओपीडी सेवाओं का पुनः उपयोग करना चाहेंगे?	☐ Yes / हाँ ☐ No / नहीं ☐ Not sure / निश्चित नहीं
14. Do you have any suggestions or comments that could help improve the waiting time or overall OPD services? क्या आपके पासऐसा कोई सुझाव या टिप्पणी है जिससे प्रतीक्षा समय या समग्र ओपीडी सेवाओंमें सुधार किया जा सके?	

## References

[1] Oche M, Adamu H (2013). Determinants of patient waiting time in the general outpatient department of a tertiary health institution in north Western Nigeria. Ann Med Health Sci Res.

[2] Mohebbifar R, Hasanpoor E, Mohseni M, Sokhanvar M, Khosravizadeh O, Mousavi Isfahani H (2013). Outpatient waiting time in health services and teaching hospitals: a case study in Iran. Glob J Health Sci.

[3] Biya M, Gezahagn M, Birhanu B, Yitbarek K, Getachew N, Beyene W (2022). Waiting time and its associated factors in patients presenting to outpatient departments at Public Hospitals of Jimma Zone, Southwest Ethiopia. BMC Health Serv Res.

[4] Langstieh AJ, Sarkar C, Wahlang JB, Marak AC, Synmon B, Jaba I (2022). A study on patient waiting time in neurology OPD of a tertiary healthcare centre and its association with patients’ demographic profile. Int J Sci Technol Res Arch.

[5] Patel R, Patel HR (2017). A study on waiting time and out-patient satisfaction at Gujarat Medical Education Research Society Hospital, Valsad, Gujarat, India. Int J Community Med Public Health.

[6] Acharya A, Kendre V, Jadhav Y, Tambe M (2023). Association between waiting time, service time and patient satisfaction in the out-patient department of a tertiary care hospital in Maharashtra. Int J Community Med Public Health.

[7] Saxena R, Sharma S, Sharda V, G N, Yadav MK (2020). A study of waiting time of patients in outpatient department of armed forces Tertiary Hospital in Northern India. J Community Health Manag.

[8] Joshi DC, Saini RS, Samant S, Bijlwan S (2023). Estimation of waiting time and consultation duration for patients in the outpatient department of radiation oncology at a tertiary care teaching hospital in Uttarakhand, India: a cross-sectional study. J Clin Diagn Res.

[9] Jain A, Mishra N, Pandey C (2016). A study to assess patient satisfaction in out patient department of a tertiary care hospital in north India. Int J Community Med Public Health.

[10] Sarwat A (2021). The effects of waiting time and satisfaction among patients visiting medical outpatient department of a tertiary care hospital. J Pak Psychiatr Soc.

[11] Xie Z, Or C (2017). Associations between waiting times, service times, and patient satisfaction in an endocrinology outpatient department: a time study and questionnaire survey. Inquiry.

[12] Zhang H, Ma W, Zhou S, Zhu J, Wang L, Gong K (2023). Effect of waiting time on patient satisfaction in outpatient: an empirical investigation. Medicine (Baltimore).

[13] Susmitha G, Sandeep K, Keerthi P (2019). Impact of waiting time for imaging tests and consultation on out-patient satisfaction at a tertiary care hospital. Int J Adv Res.

[14] Hasibuan R (2025). Hospital outpatient waiting time and patient satisfaction: an observational comparative study. Soc Med.

[15] von Elm E, Altman DG, Egger M, Pocock SJ, Gøtzsche PC, Vandenbroucke JP (2007). The Strengthening the Reporting of Observational Studies in Epidemiology (STROBE) statement: guidelines for reporting observational studies. Lancet.

[16] Thapa R, Saldanha S, Bucker N, Rishith P (2018). An assessment of patient waiting and consultation time in the outpatient department at a selected tertiary care teaching hospital. J Evol Med Dent Sci.

[17] Khan H, Singh AK (2021). A study on waiting time of the OPD patient in a multispecialty hospital. Int J Res Appl Sci Eng Technol.

[18] Sriram S, Noochpoung R (2018). Determinants of hospital waiting time for outpatient care in India: how demographic characteristics, hospital ownership, and ambulance arrival affect waiting time. Int J Community Med Public Health.

[19] Wafula RB, Ayah R (2021). Factors associated with patient waiting time at a medical outpatient clinic: a case study of University of Nairobi Health Services. Int J Innov Res Med Sci.

[20] Elemoah D, Abeasi DA, Agyei FB, Sasemame ES, Akumiah PO (2025). The waiting game: determinants and effects of OPD service delays in a Tertiary Health Facility. BMC Health Serv Res.

[21] Krishanth MDA, Fernando KES, Liyanage DH (2021). Factors contributing to increased patient waiting time at out patient department of Lady Ridgeway Hospital for Children in Sri Lanka and strategies to minimize the waiting time. Int J Sci Adv.

[22] Joshi DC, Saini RS, Bhardwaj K (2025). Patient satisfaction in the outpatient department at a tertiary care teaching hospital: a cross-sectional study. Int J Health Sci Res.

[23] Deorankar DR, Shinde V, Gudhe V (2021). Assessment of waiting time and recommend the strategies for reduction of waiting time in outpatient department at AVBRH, Wardha, India. J Pharm Res Int.

[24] Lee H, Choi EK, Min KA, Bae E, Lee H, Lee J (2022). Physician-customized strategies for reducing outpatient waiting time in South Korea using queueing theory and probabilistic metamodels. Int J Environ Res Public Health.

[25] Munavalli JR, Rao SV, Srinivasan A, van Merode GG (2020). Integral patient scheduling in outpatient clinics under demand uncertainty to minimize patient waiting times. Health Informatics J.

